# Aptamer-conjugation targets decoy-ODN-therapeutics to liver fibrosis

**DOI:** 10.1016/j.omtn.2025.102660

**Published:** 2025-08-12

**Authors:** Larissa W. Picard, Ziwen Jiang

**Affiliations:** 1 Department of Chemistry and Biochemistry, University of Texas at Dallas, Richardson, TX 75080, USA

## Main text

**Figure 1:**
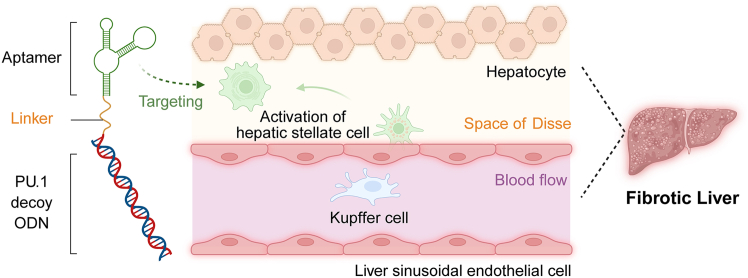
Schematic illustration of how aptamer-conjugation targets decoy oligodeoxynucleotide to the active hepatic stellate cell for liver fibrosis treatment The figure was created with BioRender.com.

Though liver fibrosis (LF) is a common condition in the world at large, there is currently no direct treatment for LF.^1^ In this study,^2^ researchers reported a potential therapeutic design for LF treatment by conjugating an aptamer (Apt-Tan) to a purine-rich box 1 decoy oligodeoxynucleotide (PU.1 decoy ODN), a therapeutic strategy that blocks gene transcription (Figure 1). The Apt-Tan interacts with unidentified membrane proteins on activated hepatic stellate cells (HSCs), enhancing the targeting capability of the Apt-Tan-PU.1 decoy ODN conjugate toward HSCs. The authors found that LF-model mice treated with the conjugate showed an increased alleviation from LF symptoms when compared to the decoy ODN alone, demonstrating the promising role of aptamer-conjugation in therapeutic targeting.

LF is a disease characterized by an excessive accumulation of the proteins from the extracellular matrix (ECM).^3^ The overproduction of ECM components is a result of the activation of HSCs upon liver injury.^1^ These injuries can be induced either by exposure to external factors (including viral hepatitis and excess alcohol intake) or by insufficient bile flow in the liver. In heathy adults, external stimuli-induced fibrosis can reverse by itself when the injury is healed, e.g., elimination of hepatitis.^1^ However, for patients with metabolic disorders such as non-alcoholic steatohepatitis (NASH), it is critical to introduce therapeutic approaches to directly mitigate fibrosis. As a representative class of treatment options, nucleic acid-based treatments allow researchers to directly manipulate the genetic activities that are responsible for fibrosis.^4^

Among the large variety of functional nucleic acids, the core design of this study is based on decoy oligodeoxynucleotides (decoy ODNs), which mimic the starting sequence of a gene and bind it to prevent transcription.^5^ PU.1 decoy ODN specifically works by binding to (and blocking) the PU.1 gene’s transcription factor binding site. Considering the contributing role of PU.1 in the activation of HSCs, inhibiting the PU.1 transcription will reduce the activation of HSCs and help with alleviating the fibrosis symptoms. The authors have attempted treatment with ODN-alone, but the ODN was not internalized by the liver cells. Instead, these ODNs were sent to and excreted by the kidney. This off-targeting effect demonstrates the need for a targeted delivery system to ensure that the ODN can reach the desired cell type (i.e., HSCs), reducing the undesired inhibitory effect when delivered to non-target cells.

Aptamers are a class of functional nucleic acids that can introduce the cell-targeting capability to decoy ODNs. Aptamers are single-stranded short RNA or DNA oligonucleotides selected by SELEX (systematic evolution of ligands by exponential enrichment) with enhanced binding affinities toward the target of interest, ranging from small molecules to whole cells.^4^ The process of cell-SELEX involves both a selection and a counter-selection step.^6^ Briefly, the selection step refines the aptamers that tightly bind to the target cell; the counter-selection focuses on the selected aptamer pool and removes the ones that bind to non-target cells. Note that a well-designed counter-selection is necessary to ensure the aptamer’s specificity. In this study, counter-selection was conducted with an assortment of non-target liver cells, including quiescent HSCs, hepatocytes, liver sinusoidal endothelial cells, and Kupffer cells. This process can therefore maximize the targeting capability of these aptamers toward active HSCs. Once the selected aptamers are conjugated to the PU.1 ODNs (Apt-Tan-PU.1 decoy ODN), the authors have shown a significant increase in targeting the decoy ODNs to the active HSCs.

In the animal studies of the report, the Apt-Tan-PU.1 decoy ODN shows promise as a treatment capable of reversing the disease symptoms of LF. As the control group, mice with CCl_4_-induced LF that did not receive the treatment had livers with a hard surface and obvious granular nodules, deposition of collagen fibers, and large amounts of cell necrosis, which are typical LF symptoms. The group that was administered with Apt-Tan-PU.1 decoy ODN had a softer surface, a significant reduction in surface nodules, and a decrease in collagen fiber deposition. After being treated with Apt-Tan-PU.1 decoy ODN, the tested HSC activation marker (α-smooth muscle actin [αSMA]) returned to the same level or lower than those of the blank group, in which no CCl_4_ was injected. Note that even though the CCl_4_-induction is one of the most frequently used methods to generate LF models, animals from this model have different pathophysiological features from patients with NASH. Unlike the patients, the CCl_4_-induced animal model cannot develop insulin resistance or become obese.^7^

The enhanced specificity of aptamers toward activated HSCs was also confirmed through their *in vivo* distributions. Apart from the inability of PU.1 decoy ODN to enter the cell on its own, a negligible amount of Apt-Tan-PU.1 decoy ODN was found in the heart, spleen, lung, and kidney of the mice. The conjugate did accumulate in the liver, with the highest level observed 1 h post-injection. Apt-Tan-PU.1 decoy ODN started to degrade 30 min post-injection and was completely degraded after 6 h. The authors noted that future optimizations with a stabilized version of Apt-Tan-PU.1 decoy ODN could be helpful. However, it is worth noting that previous attempts to stabilize oligonucleotides have resulted in a reduced affinity for the target.^8^ Alternatively, synthetic delivery carriers may need to be considered to further enhance the stability of the conjugate.

Finally, one of the most critical mechanistic understandings from this work would be how the selected aptamer enhances the cellular uptake of the conjugates. Due to the electrostatic repulsion with the net-negatively charged cell membrane, nucleic acids with a net negative charge typically do not lead to a sufficient level of cellular uptake to exhibit therapeutic effects. Through a pharmacological screening with several endocytic inhibitors,^9^ the authors demonstrated the contribution of caveolae-mediated endocytosis for the enhanced uptake of the conjugate. Besides, the cellular uptake of the aptamer-conjugate was significantly reduced in trypsin-treated cells. As trypsin is a proteolytic enzyme that cleaves a broad spectrum of cell surface proteins, it indicates that the uptake of Apt-Tan-PU.1 decoy ODN may involve multiple cell surface proteins. Future identifications on these contributing proteins would certainly provide valuable information on cell membrane-biomaterial interactions, facilitating the precise targeting of active HSCs. Several previous reports have identified the specific cell surface receptor for selected aptamers from cell-SELEX, introducing potential biomarkers on different cell types.^10^ Even though the number of cell-SELEX-identified biomarkers is limited, the fast development of omics technology may accelerate the speed of biomarker discovery.

The research progress from this report not only provides a potential therapeutic strategy against LF, but also opens new avenues for the exploration of aptamer-delivered gene therapies. A highly specific and biocompatible delivery system will certainly be helpful to increase the efficacy of macromolecular therapeutics, particularly facilitating the therapeutics to cross the cellular membrane barriers. Enhanced therapeutic targeting could inherently reduce the adverse effects of nucleic acid-based therapies. The design concept could also be applicable for the treatment of a variety of diseases, particularly when the cell surface biomarkers are identified from disease cell types.

## Acknowledgments

This work was supported by the 10.13039/100004917Cancer Prevention and Research Institute of Texas (CPRIT: RR250061).

## Declaration of interests

The authors declare no competing interests.
